# First identification of primary nanoparticles in the aggregation of HMF

**DOI:** 10.1186/1556-276X-7-38

**Published:** 2012-01-05

**Authors:** Mu Zhang, Hong Yang, Yinong Liu, Xudong Sun, Dongke Zhang, Dongfeng Xue

**Affiliations:** 1 School of Mechanical and Chemical Engineering and Centre for Energy, The University of Western Australia, 35 Stirling Highway, Perth, Western Australia, 6009, Australia; 2 School of Materials and Metallurgy, Northeastern University, Wen Hua Road, Shenyang, 110004, People's Republic of China; 3 State Key Laboratory of Rare Earth Resource Utilization, Changchun Institute of Applied Chemistry, Chinese Academy of Sciences, Ren Min Street, Changchun, 130022, People's Republic of China

**Keywords:** saccharides, carbohydrate, HMF, nanoparticles, carbonaceous spheres

## Abstract

5-Hydroxymethylfurfural [HMF] is an important intermediate compound for fine chemicals. It is often obtained via hydrothermal treatment of biomass-derived carbohydrates, such as fructose, glucose and sucrose. This study investigates the formation of carbonaceous spheres from HMF created by dehydration of fructose under hydrothermal conditions. The carbonaceous spheres, ranging between 0.4 and 10 μm in diameter, have granulated morphologies both on the surface and in the interior. The residual solution is found to contain a massive number of primary nanoparticles. The chemical structure of the carbonaceous spheres was characterised by means of FTIR and NMR spectroscopies. Based on these observations, a mechanism involving the formation and aggregation of the nanoparticles is proposed. This mechanism differs considerably from the conventional understanding in the open literature.

## Introduction

Hydrothermal treatment of saccharides (e.g. fructose, glucose, sucrose and starch) at elevated temperatures has attracted much attention in recent years for its technological and scientific interests [1-6]. In general, hydrothermal treatment of saccharides produces water-soluble organic substances and insoluble carbonaceous solids. The soluble organic substances have been the focus of early research, and understanding of the chemical reaction process and the products has been well established by earlier researchers [7,8]. In more recent years, the solid products, often referred to as humins in early studies, have attracted keen attention due to their potential for applications as functional nanomaterials or as nanotemplates for other materials [1,9-11]. Among these studies, several hypotheses have been suggested in the literature for the physical and chemical mechanisms for the formation of these carbonaceous solids, often in a spherical form. Earlier studies suggested that the carbonaceous spheres form via dehydration of saccharide molecules followed by aromatization under hydrothermal conditions. The carbonaceous spheres produced are thus expected to have a highly aromatic nucleus and a hydrophilic shell [1-5,9]. Another hypothesis proposed by Wang et al. [12] suggests that sucrose molecules form a kind of amphiphilic micelle compound under hydrothermal conditions, and as the concentration of this compound reaches a critical micelle concentration, spherical micelles develop. The carbonaceous spheres thus grow by the polymerization of sucrose molecules [12]. Yao et al. [2] proposed probably the most acceptable suggestion, in which fructose converts into 5-hydroxymethylfurfural [HMF] in the solution and then HMF monomers polycondense into nano-micro carbonaceous spheres via intermolecular dehydration. The microspheres further coalesce into larger spheres via a process analogous to emulsion coalescence. Despite the various concepts proposed, little direct experimental evidence have been reported in the literature to support these hypotheses. More recently, Hu et al. [13] published a review paper on hydrothermal processing of biomasses and pointed out: 'In the HTC process of carbohydrates, the formation process and the final material structures are rather complicated, and a clear scheme has not been reported'. This statement well summarises the current state of understanding of the products and their formation mechanisms.

To clarify this issue, we used fructose as a model precursor material to investigate the formation mechanism of the carbonaceous spheres under hydrothermal conditions. In this study, we identified for the first time the formation of primary nanoparticles, which serve as the building blocks for the micron-sized carbonaceous spheres. Based on this observation, we are able to elucidate that the formation mechanism of carbonaceous spheres is via aggregation of the primary nanoparticles.

## Experimental works

Fructose (99%, Sigma-Aldrich, Castle Hill, New South Wales, Australia) was used as the saccharide precursor for the hydrothermal treatment. The fructose was dissolved in distilled water to form a 7.5-wt.% solution. The solution was filled in a 100-ml, Teflon-lined, stainless steel autoclave to 80% full. The autoclave was placed into a preheated oven and maintained at a constant temperature ranging between 423 and 463 K for various durations up to 48 h. Carbon spheres formed were separated from the solution by centrifugation, followed by washing in distilled water and absolute ethanol for several times, and finally dried at 333 K for 24 h.

Morphology of the carbon spheres was characterised by means of scanning electron microscopy [SEM] (Zeiss 1555 instrument, Sydney, New South Wales, Australia) and transmission electron microscopy [TEM] (JEOL 3000 instrument, Sydney, New South Wales, Australia). Molecular structure of the carbon spheres was analysed by means of Fourier transform infrared [FTIR] spectroscopy (PerkinElmer Spectrum GX FTIR spectrometer, Melbourne, Victoria, Australia) with a resolution of 4 cm^-1^. Samples for FTIR analysis were prepared by mixing the sample powders with KBr (Ajax Finechem Pty. Ltd., Sydney, New South Wales, Australia) and compacting into discs. Solid-state ^13^C cross-polarisation magic angle spinning spectra were recorded with a Varian 400 MHz spectrometer (Melbourne, Victoria, Australia) with 4- or 6-mm zirconia rotors spinning at 5 kHz. A recycle delay of 2 s and a contact time of 2 ms were employed with SPINAC decoupling during acquisition. Typically, 1, 600 scans were acquired, and exponential multiplication with a line broadening of 100 Hz was applied prior to Fourier transformation.

## Results

### SEM and TEM identification of primary particles and their aggregation

**Figure 1:**
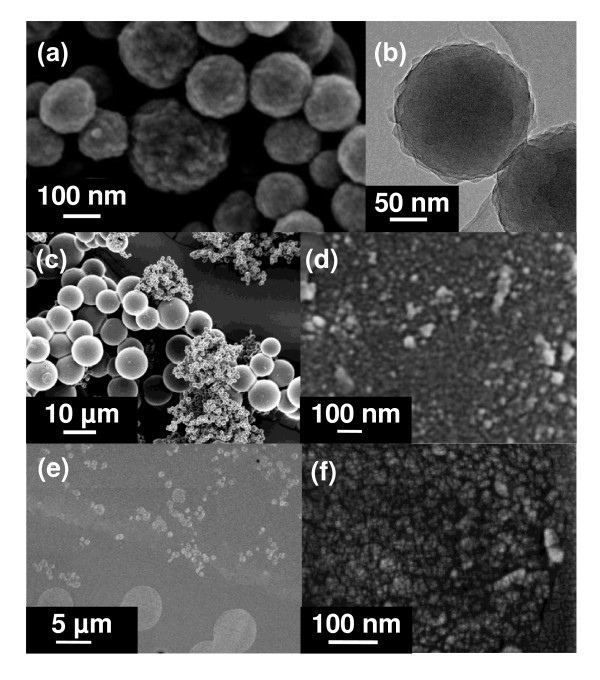
**SEM and TEM images of carbonaceous spheres, revealing details of grainy surfaces and interiors**. Carbon spheres produced at (**a**, **b**) 423 and (**c**, **d**) 453 K. (**e**, **f**) Cross-sectional views revealing the interior of the carbonaceous spheres.

Hydrothermal treatment of fructose solution in an autoclave at temperatures in the range of 423 to 463 K for different times produced carbonaceous solids in a spherical shape. Figure 1 shows SEM images of carbon spheres produced under hydrothermal conditions. Micrograph (a) shows carbon spheres synthesised at 423 K for 6 h. The spheres, typically 100 to 300 nm in diameter under these conditions, are granular on their surfaces. Micrograph (b) is a TEM image of the same sample, revealing the same features.

Hydrothermal treatment at higher temperatures produced larger, smooth and nearly perfect spheres, with diameters in the range of 0.4 to 10 μm. Micrograph (c) shows a sample treated at 453 K for 6 h. Micrograph (d) shows the surface morphology of a large, smooth sphere at high magnification. It is evident that the surface is rough and granulated. The granules are typically approximately 5 nm in size. To further examine the interior structure of the carbon spheres, the carbon sphere powders were cast into epoxy and then sliced for examination of their cross sections. Micrographs (e) and (f) show the SEM images of a sliced sample. Micrograph (e) shows a low-magnification image of the cross section of the sample, capturing both populations of the large and small spheres. Micrograph (f) shows the details of the interior of the carbonaceous sphere, revealing that the interior consisted of entirely nano-sized particles, typically approximately 5 nm.

**Figure 2:**
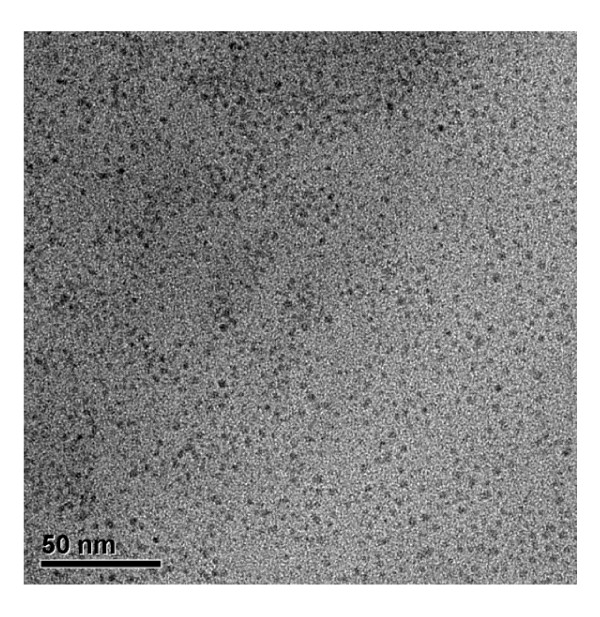
**TEM image of primary nanoparticles in a residual fructose solution after hydrothermal treatment**.

Figure 2 shows TEM images of a sample prepared from a residual fructose solution after hydrothermal treatment at 423 K for 6 h. It is seen that the residual solution contained a large population of nano-sized carbonaceous particles. These nanoparticles, hereafter referred to as primary particles, are uniform and are typically approximately 5 nm in size.

### Chemical structure of carbonaceous spheres

**Figure 3:**
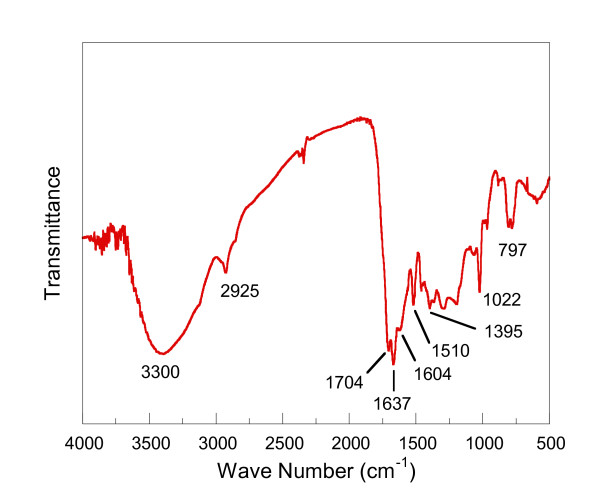
**FTIR spectrum of carbon spheres derived from fructose**.

Figure 3 shows an FTIR spectrum of carbon spheres synthesised at 453 K. The broad band at approximately 3, 300 cm^-1 ^corresponds to O-H stretching of carboxylic bonds [14]. The band at 2, 920 cm^-1 ^is due to asymmetric C-H stretching of aliphatic groups. The shoulder at 1, 704 cm^-1 ^is an indication of undissociated carbonyl groups. The vibrations at 1, 604, 1, 510 and 1, 395 cm^-1 ^are the characteristic band stretches of the five-member heteroaromatic ring with double bonds [15]. The bands at 800 to 700 cm^-1 ^may be assigned to strong hydrogen wag absorption of the five-membered ring with a CH = CH group unsubstituted [15]. This spectrum indicates that the carbon spheres are the derivatives of HMF, as evidenced by the signature five-member heteroaromatic rings. HMF is an intermediate compound formed via dehydration of fructose under hydrothermal conditions, as reported by Baccile et al. [16].

**Figure 4:**
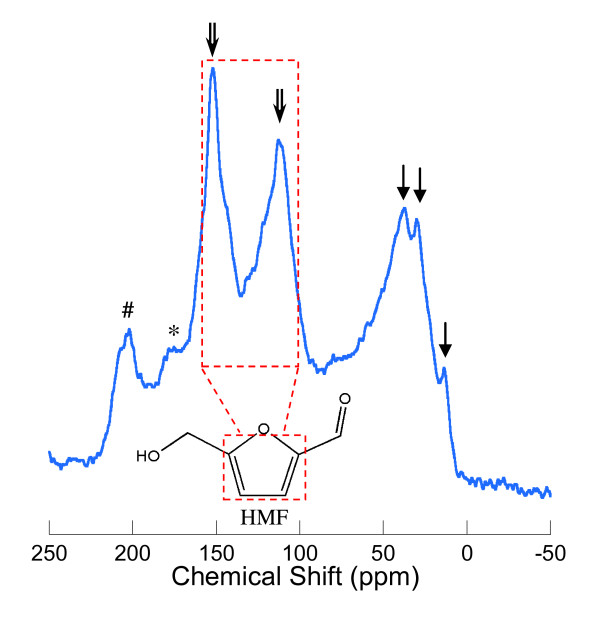
**Solid state ^13^C NMR spectrum of carbon spheres produced by fructose**. Single arrows indicate aliphatic groups. Double arrows in dash box indicate furanic ring. Asterisk indicates C = O group.

Figure 4 shows a solid-state ^13^C nuclear magnetic resonance [NMR] spectrum of carbonaceous spheres synthesised at 453 K. The peak at 13.55 is attributed to mobile CH_3 _groups. The peaks at 30.09 and 38.60 ppm (indicated by the single arrows) are characteristic of *sp*^3 ^carbon atoms, indicating the presence of aliphatic species in the sample. In reference to our solution ^13^C NMR analysis, these peaks are assigned to an embedded levulinic acid [16]. The peaks at 111.87 and 151.38 ppm (indicated by the double arrows) are associated with O-C = CH and O-C = CH sites on the furan ring, respectively [16,17]. These peaks are attributed to a heterocyclic aromatic compound - furan, indicating the presence of HMF aromatic rings [17]. The broad peak at 170 to 180 ppm (marked by *) is attributed to C = O groups in ketones and aldehydes from HMF, and embedded levulinic and formic acids [16]. The peak at 200.75 ppm (marked by #) is a spinning side band. This spectrum demonstrates that the carbonaceous spheres are composed of cross-linked furan rings derived from HMF, rather than graphene-type species or saccharide molecular link, as claimed in the literatures [1-4,9]. This evidence, together with the FTIR analysis, further suggests that HMF, rather than fructose, is the feedstock of carbonaceous spheres. Elemental analysis of the solid carbon spheres showed that the spheres contained 65.7 wt.% C, 4.3 wt.% H and 30.0 wt.% O, corresponding to a molecular formula of C_6_H_0.59_(H_2_O)_2.06_. This corresponds to a loss of 0.94 H_2_O per molecular unit of HMF.

## Discussion

**Figure 5:**
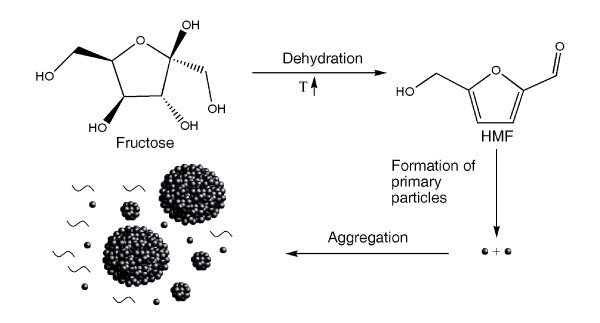
**Schematic illustration of the formation mechanism of carbonaceous spheres from fructose under hydrothermal condition**.

From all observations and analyses obtained in this study, we propose the following hypothesis as the formation mechanism of carbonaceous spheres from fructose under hydrothermal conditions, as schematically illustrated in Figure 5. Under hydrothermal conditions, fructose undergoes dehydration to form HMF. This has been proven in the literature [8,18]. HMF monomer has active functional groups, such as the hydroxyl terminal. This renders the HMF monomer the ability to polycondense via intra-molecular dehydration through reactions between the hydroxyl and H-terminals of different HMF monomers to form cross-linked furanic species. The continued growth in size of the cross-linked furanic species eventually results in the precipitation of the molecular clusters out of the solution into the primary carbonaceous nanoparticles. These primary nanoparticles, having inherited the functional groups of HMF on their surfaces, may continue to aggregate via the same polycondensation reactions as those causing the formation of the primary particles, leading to the formation of the large, near carbonaceous spheres. This concept, supported by the direct experimental evidence and the known chemistry of HMF, differs significantly from the conventional hypotheses in the open literature.

## Conclusions

In this study, carbonaceous spheres were produced from fructose under hydrothermal conditions. The experimental evidence clearly demonstrates that the carbonaceous spheres are formed as aggregates of nanoparticles. TEM observation of residual solutions after hydrothermal treatment provides the direct and first evidence of the presence of these primary nanoparticles. Based on these observations, a new mechanism for the formation of carbonaceous spheres from saccharides has been proposed. The mechanism involves three steps, including dehydration of fructose into HMF, polycondensation of HMF monomers into primary particles via intra-molecular dehydration and aggregation of primary nanoparticles in carbonaceous spheres. This mechanism differs significantly from the conventional understanding in the open literature.

## Competing interests

The authors declare that they have no competing interests.

## Authors' contributions

MZ carried out the experimental work and drafted the manuscript. HY is the guiding scientist who supervised the research and contributed to the scientific argument and in drafting of the manuscript. YNL is the co-supervisor who participated in the data analysis and drafting of the manuscript. XDS contributed in the planning of the experimental program and in discussing of carbon sphere formation mechanism. DKZ contributed in the analysis of FTIR experimentation and discussion of carbon sphere formation mechanism. DFX contributed in the theory of primary particles and the discussion of the chemical structure of carbon spheres. All authors read and approved the final manuscript.

## References

[B1] Wang Q Li H Chen L Huang X Monodispersed hard carbon spherules with uniform nanopores Carbon 2001 39 2211 2214 10.1016/S0008-6223(01)00040-9

[B2] Yao CH Shin YS Wang LQ Windisch CF Samuels WD Arey BW Wang CM Risen WM Jr Exarhos GJ Hydrothermal dehydration of aqueous fructose solutions in a closed system J Phys Chem C 2007 111 15141 15145 10.1021/jp074188l

[B3] Sevilla M Fuertes AB Chemical and structural properties of carbonaceous products obtained by hydrothermal carbonization of saccharides Chem Eur J 2009 15 4195 4203 10.1002/chem.200802097 19248078

[B4] Sevilla M Fuertes AB The production of carbon materials by hydrothermal carbonization of cellulose Carbon 2009 47 2281 2289 10.1016/j.carbon.2009.04.026

[B5] Titirici MM Antonietti M Baccile N Hydrothermal carbon from biomass: a comparison of the local structure from poly- to monosaccharides and pentose/hexoses Green Chem 2008 10 1204 1212 10.1039/b807009a

[B6] Chen C Sun X Jiang X Niu D Yu A Liu Z Li J A two-step hydrothermal synthesis approach to monodispersed colloidal carbon spheres Nanoscales Res Lett 2009 4 971 976 10.1007/s11671-009-9343-5 PMC2894148 20596393

[B7] Romάn-Leshkov Y Chheda JN Dumesic JA Phase modifiers promote efficient production of hydroxymethylfurfural from fructose Science 2006 312 1933 1937 10.1126/science.1126337 16809536

[B8] Chheda JN Romάn-Leshkov Y Dumesic JA Production of 5-hydroxymethylfurfural and furfural by dehydration of biomass-derived mono- and poly-saccharides Green Chem 2007 9 342 350 10.1039/b611568c

[B9] Sun XM Li YD Colloidal carbon spheres and their core/shell structures with noble-metal nanoparticles Angew Chem Int Ed Engl 2004 43 597 601 10.1002/anie.200352386 14743414

[B10] Titirici MM Antonietti M Thomas A A generalized synthesis of metal oxide hollow spheres using a hydrothermal approach Chem Mater 2006 18 3808 3812 10.1021/cm052768u

[B11] Ji X Huang X Liu J Jiang J Li X Ding R Hu Y Wu F Li Q Carbon-coated SnO_2 _nanorod array for lithium-ion battery anode material Nanoscales Res Lett 2010 5 649 653 10.1007/s11671-010-9529-x PMC2894033 20672094

[B12] Wang Q Li H Chen L Huang X Novel spherical microporous carbon as anode material for Li-ion batteries Solid State Ionics 2002 152-153 43 50

[B13] Hu B Wang K Wu L Yu S Antonietti M Titirici MM Engineering carbon materials from the hydrothermal carbonization process of biomass Adv Mater 2010 22 813 828 10.1002/adma.200902812 20217791

[B14] Muscolo A Sidari M Attina E Francioso O Tugnoli V Nardi S Biological activity of humic substances is related to their chemical structure Soil Sci Soc Am J 2007 71 75 85 10.2136/sssaj2006.0055

[B15] Colthup NB Daly LH Wiberley SE Introduction to Infrared and Raman Spectroscopy 1975 New York: Academic Press

[B16] Baccile N Laurent G Babonneau F Fayon F Titirici MM Antonietti M Structure characterization of hydrothermal carbon spheres by advanced solid-state MAS ^13^C NMR investigations J Phys Chem C 2009 113 9544 9554

[B17] Memory JD NMR of Aromatic Compounds 1982 North Carolina: John Wiley & Sons

[B18] Kuster BFM van der Baan HS The influence of the initial and catalyst concentrations on the dehydration of D-fructose Carbohydrate Res 1977 54 165 176 10.1016/S0008-6215(00)84806-5

